# Estimating Summer Maize Biomass by Integrating UAV Multispectral Imagery with Crop Physiological Parameters

**DOI:** 10.3390/plants13213070

**Published:** 2024-10-31

**Authors:** Qi Yin, Xingjiao Yu, Zelong Li, Yiying Du, Zizhe Ai, Long Qian, Xuefei Huo, Kai Fan, Wen’e Wang, Xiaotao Hu

**Affiliations:** 1 Key Laboratory of Agricultural Soil and Water Engineering in Arid and Semiarid Areas, Ministry of Education, Northwest AF University, Yangling 712100, China; yq2023055852@nwafu.edu.cn (Q.Y.); yxj0204@nwafu.edu.cn (X.Y.); lizl@nwafu.edu.cn (Z.L.); dyysweet@nwafu.edu.cn (Y.D.); azz@nwafu.edu.cn (Z.A.); qianlong@nwafu.edu.cn (L.Q.); hxf@nwafu.edu.cn (X.H.); 2022050889@nwafu.edu.cn (K.F.); huxiaotao11@nwsuaf.edu.cn (X.H.); 2 College of Water Resources and Architectural Engineering, Northwest AF University, Yangling 712100, China

**Keywords:** AGB, plant height, UAV, deep learning model

## Abstract

The aboveground biomass (AGB) of summer maize is an important indicator for assessing crop growth status and predicting yield, playing a significant role in agricultural management and decision-making. Traditional on-site measurements of AGB are limited, due to low efficiency and a lack of spatial information. The development of unmanned aerial vehicle (UAV) technology in agriculture offers a rapid and cost-effective method for obtaining crop growth information, but currently, the prediction accuracy of summer maize AGB based on UAVs is limited. This study focuses on the entire growth period of summer maize. Multispectral images of six key growth stages of maize were captured using a DJI Phantom 4 Pro, and color indices and elevation data (DEM) were extracted from these growth stage images. Combining measured data such as summer maize AGB and plant height, which were collected on the ground, and based on the three machine learning algorithms of partial least squares regression (PLSR), random forest (RF), and long short-term memory (LSTM), an input feature analysis of PH was carried out, and a prediction model of summer maize AGB was constructed. The results show that: (1) using unmanned aerial vehicle spectral data (CIS) alone to predict the biomass of summer maize has relatively poor prediction accuracy. Among the three models, the LSTM (CIS) model has the best simulation effect, with a coefficient of determination (R^2^) ranging from 0.516 to 0.649. The R^2^ of the RF (CIS) model is 0.446–0.537. The R^2^ of the PLSR (CIS) model is 0.323–0.401. (2) After adding plant height (PH) data, the accuracy and stability of model prediction significantly improved. R^2^ increased by about 25%, and both RMSE and NRSME decreased by about 20%. Among the three prediction models, the LSTM (PH + CIS) model had the best performance, with R^2^ = 0.744, root mean square error (RSME) = 4.833 g, and normalized root mean square error (NRSME) = 0.107. Compared to using only color indices (CIS) as the model input, adding plant height (PH) significantly enhances the prediction effect of AGB (aboveground biomass) prediction in key growth periods of summer maize. This method can serve as a reference for the precise monitoring of crop biomass status through remote sensing with unmanned aerial vehicles.

## 1. Introduction

Maize is the largest crop in China, with an annual output accounting for 23% of the global total. However, due to the limited cultivated area and gradually increasing food demand, it is urgent to improve the production efficiency of maize and achieve higher grain yields [1]. Aboveground biomass (AGB) is an important indicator for the real-time monitoring of plant growth. Biomass data will change correspondingly with plant growth. Thus, the real-time monitoring of crop biomass can lead to a better grasp of crop growth [2]. It can detect problems in time to prevent issues and provide corresponding solutions to better achieve the goal of high yield [3]. Traditional AGB data acquisition relies on destructive sampling, which is time-consuming and laborious and difficult to extend to large-area crop planting. Unmanned aerial vehicle remote sensing can better solve such problems. Unmanned aerial vehicles can be used to obtain multispectral images of crops and extract data such as texture, plant height, and the spectra of crops [4]. They overcome the problems of high cost and low efficiency of traditional biomass monitoring methods and provide technical means of realizing the rapid and accurate real-time dynamic monitoring of biomass on a large scale [5].

At present, satellite remote sensing and unmanned aerial vehicle (UAV) remote sensing are usually used to obtain remote sensing data. Through different technical means and by using different cameras, image data such as multispectral, hyperspectral, and ultraspectral images can be obtained. The advantage of multispectral images is that the remote sensing data of different spectral bands and richer image data than from traditional methods can be obtained, which is convenient for better identifying ground-object images [6]. In recent years, research on quantitative biomass inversion using unmanned aerial vehicle multi-source remote sensing has become more and more extensive [7,8]. Liu et al. [9] used remote sensing data to predict the biomass of potatoes. Xiao et al. [10] used remote sensing data, combined with deep learning, to monitor the growth of maize. The above traditional methods use spectral data for inversion; biological parameter data are not combined in the research. Zhai et al. [7] estimated maize above-ground biomass using unmanned aerial vehicle-based multi-source sensor data and SPAD values. Wang et al. [11] successfully predicted the biomass of wheat by combining plant height with spectral data. Looking at past studies, there are signs indicating that combining crop parameters with spectral data can improve the prediction accuracy of crop biomass calculations and offers a new technique for predicting the biomass of summer maize by combining crop parameters [12,13].

According to previous research results, the correlation between spectral information extracted by traditional methods and plant biomass will change with changes during different growth periods of corn [14]. Using a single correlation to predict biomass throughout the growth period is not accurate. Spectral features also have certain limitations in phenotypic monitoring. In the reproduction stage of crops during the later growth period, spectral saturation is prone to occur. If data after spectral saturation are used to establish a model, the prediction result of the model is inaccurate and will lead to errors in practical applications. Since spectral saturation means that part of the spectral information is truncated and a complete and accurate spectral signal cannot be obtained, this issue will destroy the linear relationship between spectral intensity and crop data, making it impossible to accurately predict data [15]. In most of the current studies, only the spectral data obtained by unmanned aerial vehicles are used for prediction, and biomass prediction is not carried out in different growth periods. To solve this problem, multiple key growth periods of summer maize were monitored, and the data from each growth period were independently analyzed. Unlike previous related studies, it is hoped that this method can improve the prediction accuracy of biomass [16]. After obtaining accurate spectral data for each key growth period, a deep learning model was established to perform nonlinear correction on possible nonlinear situations in the data, so as to avoid the impact of spectral saturation as much as possible and accurately predict crop biomass [17].

The crop PH value is an important indicator reflecting the growth status of crops. Studies have proved that there is a good correlation between PH and AGB [18,19]. With the development of remote sensing technology, the methods for obtaining PH data are gradually enriched. Traditional sensors, such as LiDAR and ultrasonic sensors, can directly extract elevation information, but they are often accompanied by some defects, such as a high cost, difficult data processing, and being easily affected by factors such as distance and environment [20]. For this paper, we extracted the crop surface model (CSM) using an unmanned aerial vehicle multispectral camera. CSM refers to the difference between the digital surface model (DSM, the height of vegetation on bare ground) and the corresponding digital elevation model (DEM, the height of the bare ground). After data processing, the crop PH value can finally be obtained through unmanned aerial vehicles [21]. Unmanned aerial vehicle remote sensing technology offers significant advantages such as a short monitoring cycle, strong timeliness, and easy coverage of large areas. It has become an important means for crop monitoring and disaster prevention. Compared with the cumbersome sensor measurement techniques in the past, it is more time-saving and labor-saving [22].

This paper takes summer corn in the Baojixia Irrigation District as the research object. For this purpose, multispectral images of corn at different growth stages were acquired using a DJI P4 unmanned aerial vehicle. Based on a traditional remote sensing data technology processing method, input feature analysis of the PH data was carried out first, and then three regression algorithm models with fitting capabilities were successfully constructed [23]. As crop growth is a dynamic process, data collection and analysis were carried out for six key growth periods of summer corn [24]. Combining long short-term memory, random forest, and partial least squares regression models to verify whether adding PH value makes a positive contribution to improving the accuracy and stability of summer corn biomass prediction, and establishing whether it has a certain universality in improving the accuracy and stability of different prediction models, this research aims to provide an effective method for better monitoring crop biomass.

## 2. Materials and Methods

### 2.1. Study Area

The test area is set in the agricultural demonstration area of Wugong County, Shaanxi Province, China (34°351′ N, 108°056′ E). The local climate is the semi-arid and semi-humid climate of the northwest region. The average temperature during the test period is about 33 °C. Rainfall in the area is mainly concentrated from July to September. The annual average precipitation is about 635 mm. The soil in the test area is clay loam. The field’s continuous moisture content of the 0–100 cm soil layer is 23–26%. The dry bulk density of the soil is 1.44 g/cm^3^ and the pH value is 8.14. The buried depth of groundwater is such that upward water replenishment can be ignored.

**Figure 1:**
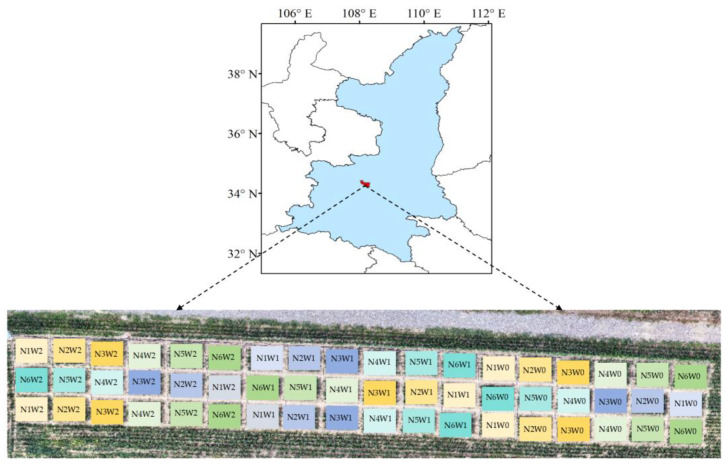
Summer maize experiment conducted in Xianyang City, Shaanxi Province, China.

A total of 54 plots were set up in the test area, and three water treatments and six nitrogen treatments were set up. These included: rain-fed (W0), deficit irrigation (W1: 60–70% of field water holding capacity), and full irrigation (W2: 90–100% of field water-holding capacity); N1 (0 kg·ha^−1^), N2 (80 kg·ha^−1^), N3 (160 kg·ha^−1^), N4 (240 kg·ha^−1^), N5 (320 kg·ha^−1^), and N6 (400 kg·ha^−1^). These were applied twice in the whole growth period at the sowing (50%) and jointing stages (50%). The test plots are shown in Figure 1.

### 2.2. Data Acquisition and Processing

**Figure 2:**
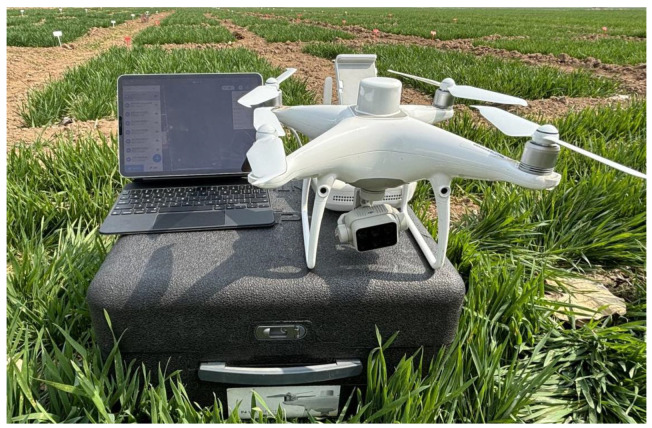
The DJI P4 Multispectral used in the experiment.

**Figure 3:**
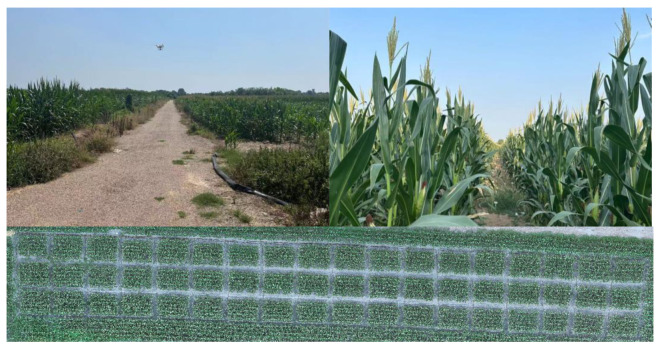
Collecting maize remote sensing data using multispectral drones and visible light images after splicing is completed.

Multispectral image acquisition was conducted, as can be seen from Figure 2 and Figure 3. We chose to carry out image acquisition on a clear and cloudless day from 9:00 to 13:00. The flight altitude of the multispectral unmanned aerial vehicle was 20 m. The course overlap rate of the unmanned aerial vehicle route was set to 75%, and the side overlap rate was 80%. A total of 6 routes were set up for the experiment to ensure full coverage of the experimental area. Since the plot size of the experimental plot was 4 m by 5 m, images were obtained by shooting at a height of 20 m. At this range, the ground resolution is 1.1 cm per pixel; under this experimental scheme, the best image data could be obtained. To prevent the unmanned aerial vehicle image from shifting, 8 ground control points (GCPS) were set up, and the coordinates of these points were measured by RTK.

The multispectral images were obtained using a DJI P4 Multispectral (DJI Technology Company Ltd., Shenzhen, China). It is equipped with six 1/2.9-inch CMOS sensors, including a color sensor for visible light imaging and five monochrome sensors for multispectral imaging. Its center wavelengths are 450 nm (B), 560 nm (G), 650 nm (R), 730 nm (RE), and 840 nm (NIR). The collected MS images were then spliced and radiometrically corrected by Pix4 Dmapper and ENVI 5.3, the DN value of the original image was converted into reflectance, and the reflectance was extracted by dividing the different ROIs.

### 2.3. Acquisition of Ground Data

Ground sampling and multispectral remote sensing data acquisition were carried out simultaneously, after collecting multispectral data at various growth stages of summer maize in 2022 and 2023 during multiple key growth periods of maize. For each test plot, two maize plants were chosen, and the PH values were measured with a ruler. Then, destructive sampling was carried out. The plant samples were divided into different parts (leaves, stems, and ears) to measure the fresh weight. After drying in an oven at 75 °C until they reached a constant weight, the dry weight of each part was measured. The AGB of each growth period was obtained by summing up the dry weights of each part [25]. During the entire growth period, a total of 648 plant samples were collected.

### 2.4. Vegetation Index Extraction

The UAV’s RGB image is a spatial image composed of three colors: red, blue, and green. The three components have a good correlation with plant AGB. Working according to previous biomass prediction studies, this study selected 17 CISs with relatively high and extensive correlations (Table 1) for AGB estimation. ENVI 5.6 software (Exelis Visual Information Solutions, Boulder, CO, USA) was used to classify plants, etc., and remove the background soil. The red, green, and blue color component images will be represented by R, G, and B. Referring to the vegetation coefficients used in past studies [26,27], we selected a part of the vegetation coefficients for correlation analysis and selected the vegetation coefficients shown in Table 1.

### 2.5. Plant Height Extraction

We obtained crop elevation data using unmanned aerial vehicles and collected bare land elevation (DEM) data before sowing. In the different growth periods, we used unmanned aerial vehicles to obtain crop surface data (CSM). After establishing the differences between the two, we used the mean value method to extract the average PH value from the CSM of each plot. We then brought the PH value data into the mathematical model and used 1/3 as the verification set to verify the PH value of the plants.

### 2.6. Model Selection

Partial least squares regression (PLSR), random forest (RF), and long short-term memory (LSTM) are three different models. Biomass prediction using CIS image data alone was compared with the prediction of summer maize biomass by combining PH and CIS. The added PH data were analyzed for input features to study the improvement seen in the accuracy of biomass estimation in important growth periods of summer maize after adding PH data [39].

The core idea of partial least squares regression (PLSR) is to find new orthogonal projection directions (principal components) so that there is the maximum covariance between the projected dependent variable and the independent variable, and then establish a prediction model [40]. Unlike principal component regression (PCR), which simply reduces the dimension of independent variables, PLSR simultaneously considers the correlation between dependent variables and independent variables in the process of dimension reduction, in order to maximize the prediction performance while reducing the dimension [41].

The random forest (RF) model is created by constructing multiple decision trees and combining multiple decision trees together. Each time, the data set is randomly selected as a replacement, and some features are randomly selected as inputs at the same time [42]. For a variety of data, the random forest model can produce classifiers with high accuracy, can handle a large number of input variables, and also gives an estimate of the importance of the variables.

The algorithm principle of long short-term memory (LSTM) is mainly reflected in its ingenious gated mechanism design. The forgetting gate allows the model to selectively “forget” information from a past state according to the current input; the input gate is responsible for screening important information in the input at the current moment and integrating it into the new candidate state; finally, the output gate determines what information should be output as the hidden state and passed to subsequent layers or used as the model output [43]. This design enables LSTM to capture long-term dependencies while avoiding the problem of gradient vanishing or explosion, thereby realizing the effective modeling of long-distance dependencies in time series data.

### 2.7. Model Construction and Accuracy Evaluation

After referring to past studies, two-thirds of the total sample was used as the training set (*n* = 432), and one-third was used as the verification set (*n* = 216) to construct a biomass estimation model for each growth period of summer maize. The verification set was only used to verify whether the model had good predictive ability and had no impact on model construction.

In this study, the coefficient of determination (R^2^), root mean square error (RMSE), and normalized root mean square error (NRMSE) are used to evaluate the accuracy of different models for predicting summer maize biomass. The closer R^2^ is to 1 and the closer RMSE and NRMSE are to 0, the smaller the error between the measured value and the predicted value, and the higher the prediction accuracy of the model. The calculation formulas are as follows:

$$R^{2}=1-\frac{{\sum_{i=1}^{n}\left(X_{i}-Y_{i}\right)}^{2}}{{\sum_{i=1}^{n}\left(X_{i}-\bar{X}\right)}^{2}}\;RMSE=\sqrt{\frac{1}{n}\sum_{i=1}^{n}{\left(Y_{i}-X_{i}\right)}^{2}}\;NRMSE=\frac{RMSE}{\bar{X}}$$

where n is the total number of samples, ‘X_i_’ is the measured value of sample i, Y_i_ is the estimated value of sample i, and ‘X’ is the mean value of the measured values of the samples.

## 3. Results

### 3.1. Feature Selection and Analysis

**Figure 4:**
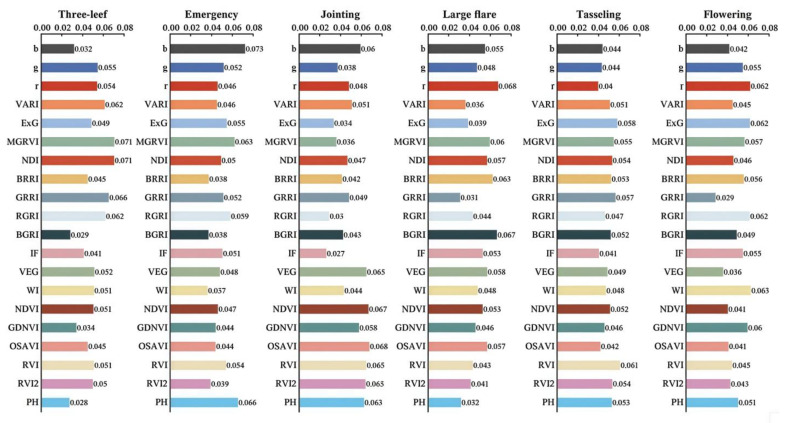
The analysis results regarding the importance of input characteristics in each growth period.

The importance of input features in different growth periods of summer maize is shown in Figure 4. Before inputting the data into the model, input feature analysis was carried out. The weights of the input data in different growth periods were all different. For example, in the jointing stage, the optimized soil-adjusted vegetation index (OSAVI) was 0.068, accounting for the largest proportion; however, in the flowering stage, it was only 0.041, accounting for a relatively small proportion. Similarly, the normalized difference vegetation index (NDI) was 0.071 in the three-leaf stage, accounting for the largest proportion; but in the flowering stage, it was only 0.046, accounting for the smallest proportion. The input feature analysis results of the added plant height (PH) data show that as an input variable, plant height showed relatively high proportions in the seedling emergence stage, flare opening stage, tasselling stage, and flowering stage of crops, which were 0.066, 0.063, 0.053, and 0.051, respectively, this being relatively high among all input variables in the corresponding periods. Thus, PH plays an important role in improving prediction accuracy. In other growth periods, plant height still has a positive effect on improving the accuracy of deep learning models.

### 3.2. Biomass Prediction Based on Vegetation Indices

**Figure 5:**
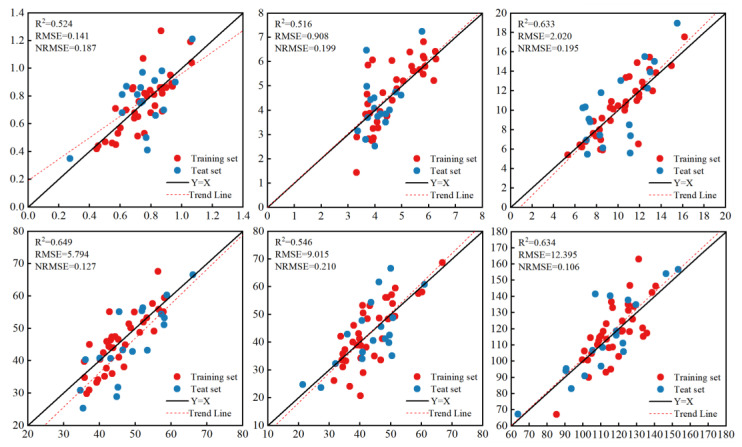
The prediction accuracy of the LSTM model at different growth stages when only vegetation indices are used.

The accuracy of biomass prediction based on vegetation indices varies significantly across the different growth stages. As the growth period progresses, the prediction accuracy of different models is slightly improved. As shown in Figure 5, among all the inversion models, the LSTM model had the best prediction effect on the flare opening stage and tasseling stage of summer maize. The R^2^ values were 0.649 and 0.634, respectively, the RMSE values were 5.794 g and 12.395 g, respectively, and the NRMSE values were 0.127 and 0.106, respectively.

As shown in Table 2, among the three models, the PLSR model has the worst inversion effect when using CIS alone. The R^2^ and NRMSE are 0.323–0.401 and 0.142–0.256, respectively. The accuracy of biomass prediction using only a single-factor CIS is still poor.

### 3.3. Biomass Prediction Combining Plant Height and Vegetation Indices

**Figure 6:**
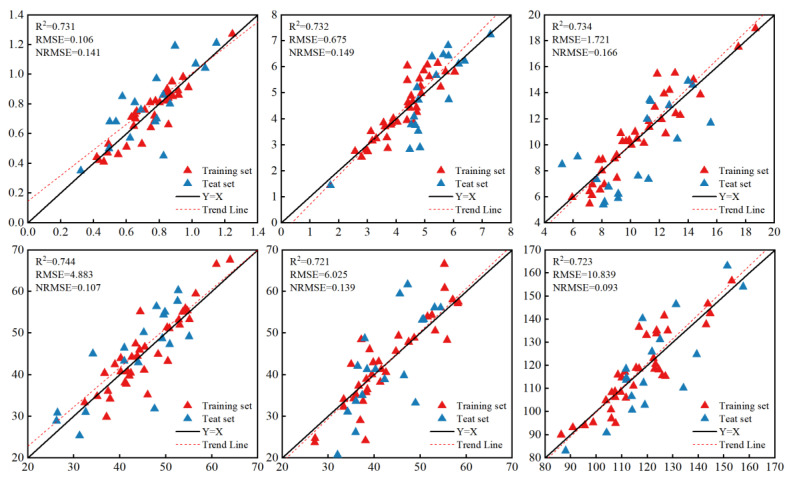
The prediction accuracy of the LSTM model at different growth stages when both vegetation coefficients and plant height are used.

The biomass prediction accuracy of all models at various growth stages significantly improved after incorporating plant height data. As shown in Table 3 and Figure 6, the LSTM model is the best one. In all growth period data, the prediction accuracy of all models was greater than 0.7. Among the models, the highest prediction accuracy was for the flare opening stage of summer maize, with R^2^ being 0.744, RSME being 4.833, and NRSME being 0.107. Considering the three estimation models, the LSTM model has the highest prediction accuracy and is the most stable.

### 3.4. Comparison of Different Model Performances

**Figure 7:**
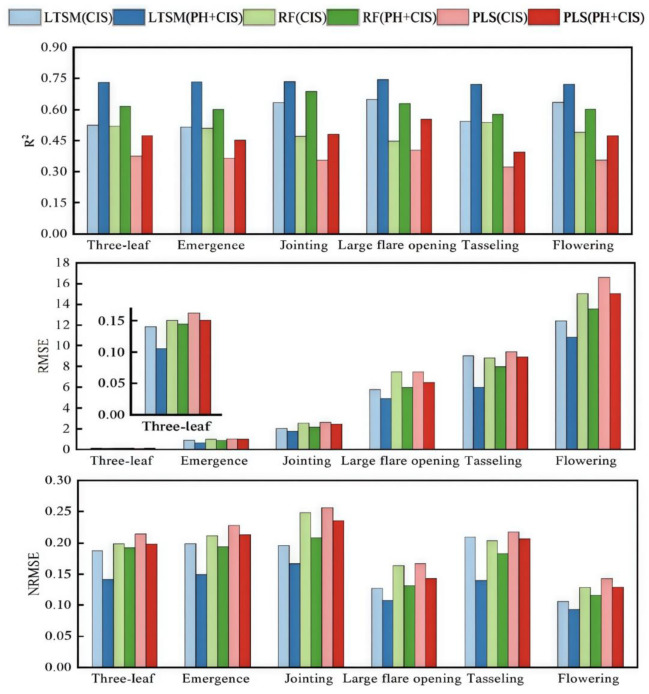
Comparison of the results of R^2^, RMSE, and NRMSE for the different models in different growth periods.

The prediction accuracy of the different models for calculating biomass during the six key growth stages of summer maize is shown in Figure 7. From the perspective of the change in the coefficient of determination R^2^, simply using CIS data had a low prediction accuracy for AGB. This is especially true with the PLSR model, where R^2^ was mostly lower than 0.4, and the prediction accuracy was poor. After adding the PH data, the prediction effect significantly improved. The R^2^ of all three models showed a significant increase after fitting with the PH data added, and this improvement ran through all key growth periods. The R^2^ of the PLSR model increased by about 29%, and the RMSE reduced by about 10%. The R^2^ of the RF and LSTM models increased by about 25%, and the RMSE reduced by about 14% and 25%, respectively. Among the three models, the LSTM model performed the best. The R^2^ was between 0.721 and 0.744, the RMSE was between 0.106 and 10.839, and the NRMSE was between 0.093 and 0.166. The fluctuation range was small, and the data-fitting effect was relatively stable.

## 4. Discussion

### 4.1. Advantages of Combining Crop Parameters

Simply using image data to monitor crop biomass and thereby determine the crop growth state is less efficient, and it is difficult to achieve an ideal effect. When the three models used in this paper only used CIS to predict biomass, the model’s prediction results were not ideal. The accuracy is poor and not stable enough, and it cannot meet the need to accurately predict biomass when only relying on image data. For the three models used in this study, the changes in modeling accuracy of the prediction model based on CIS + PH and of the AGB prediction model based on CIS were compared and analyzed. After the three models were combined with the PH data for prediction, the prediction accuracy was significantly improved. R^2^ was significantly increased, and both RMSE and NRMSE decreased. This strongly indicates that adding PH data improves the fitting effect of the summer maize biomass prediction model in different growth periods, reduces the prediction error, and improves the prediction accuracy.

However, the plant height of crops in different growth periods also changes. The plant height value is an important indicator for the growth and development of summer maize. The dynamic changes in plant height data reflect the developmental ability of summer maize to a certain extent and show the positive growth of summer maize. The test results indicate that plant height (PH) information can be an important environmental factor for the biomass estimation of summer maize in different growth periods and can improve the accuracy of monitoring summer maize biomass [44]. The research of Liu et al. [26] proves that adding plant height values can improve the aboveground biomass (AGB) estimation model of crops in different growth periods. Secondly, multivariate features inherently contain the spatiotemporal changes of summer maize populations and are always more predictive for biomass prediction than a single variable. The previous study by Li et al. [45] also mentions that different canopy height estimations can improve the accuracy of biomass prediction. However, there are many influencing factors when monitoring biomass using remote sensing technology, and the plant height changes of different crops in different growth periods are also different. Compared with previous studies, this study has collected and analyzed the data for six key growth periods of summer maize. Referring to past research results, there are differences in the recorded plant height data for the same crop in different growth periods and in the data for different growth periods of the same crop. For the monitoring of crop biomass, each growth period should be processed separately to obtain the best biomass prediction effect. In future research, factors such as growth periods, different crop types, and their growth environments should be accurately divided up to improve the accuracy and stability of prediction and provide reliable new technologies for current agricultural development.

### 4.2. Efficiency of Deep Learning Models

When dealing with large amounts of data, deep learning models can learn more general and comprehensive patterns and laws. In the face of nonlinear data situations, with its multi-layer neural network structure, a deep learning model can dig out the complex relationships therein. For example, models such as RF and LSTM can continuously optimize their parameters in the face of small changes and uncertainties in the data, thereby significantly improving the accuracy and precision of prediction [46]. The LSTM model has the highest accuracy. This is because, compared with other traditional machine learning models, the LSTM model solves the problem of long-term dependence when processing data by introducing a gating mechanism and can better handle long-term dependencies in long-sequence data. The significant defect of traditional machine learning methods is that when dealing with a large amount of data without obvious collinearity, it is necessary to manually perform feature selection or dimensionality reduction on the data in advance. Due to its own characteristics, the deep learning model is less sensitive to the collinearity of feature variables and can process some data without obvious linear relationships [47].

This paper uses the long short-term memory network (LSTM), random forest (RF), and partial least squares regression (PLSR) algorithms to construct a summer maize biomass prediction model. The results show that compared with traditional machine learning algorithms, when using the optimal data set, all three deep learning models exhibit good fitting effects. Previous researchers, e.g., Wang et al. [11], successfully predicted wheat biomass by combining wheat plant height data with machine learning models. Shu et al. [48] successfully predicted corn biomass by combining corn canopy data with machine learning models. Past research indicates that deep learning models help overcome the limitations of prediction from a single data source. Compared with traditional methods that rely only on spectral data, the prediction effect when combined with biological parameter data is significantly better. In this paper, combining corn plant height data with spectral data and inputting it into the deep learning model effectively improves the prediction accuracy for summer maize biomass. This strongly proves the feasibility of biomass prediction combined with biological parameters and also provides a new method for summer maize biomass monitoring.

## 5. Conclusions

This study obtained a crop surface model and high-resolution digital canopy image of summer maize through the use of multispectral unmanned aerial vehicles. The PH data of summer maize was extracted, and three summer maize biomass estimation models were constructed, based on CIS images and the addition of PH data.

Deep learning models perform well in terms of estimation accuracy. Compared with traditional machine learning models, they have better fitting effects when dealing with large amounts of data. In this paper, the prediction accuracy of the LSTM model is generally high, and the fitting effect is good. The optimal verification set has an R^2^ of 0.744, an RMSE of 4.833 g, and an NRSME of 0.107.

Compared with using only CIS for prediction, after adding PH data, the prediction accuracy and stability of all models are significantly improved. Through the comparison and analysis of the results of the three models, adding PH value provides positive help in improving the accuracy and stability of predicting summer maize biomass, and lends a certain universality for improving the accuracy and stability of different prediction models. It also provides new technical possibilities for monitoring crop AGB.

## Figures and Tables

**Table 1:** Vegetation coefficients.

Color Indices	Formula	Reference
r	R/(R + G = B)	
g	G/(R + G = B)	
b	B/(R + G = B)	
VRAI	(g − r)/(g + r − b)	[28]
ExG	2g − r − b	[29]
MGRVI	(g^2^ − r^2^)/(g^2^ + r^2^)	[30]
NDI	(r-g)/(r + g + 0.01)	[29]
BRRI	3g − 2.4r − b	[29]
GRRI	(g^2^ − br)/(g^2^ + br)	[31]
RGRI	R/G	[32]
BGRI	B/G	[29]
NPCI	(R − B)/(R + B)	[33]
IF	(2G − R − B)/(G − B)	[30]
VEG	G/(R0.667 × B0.333)	[34]
WI	(G − B)/(R − G)	[29]
NDVI	NDVI = (NIR − R)/(NIR + R)	[35]
GDNVI	GNDVI = (NIR − G)/(NIR + G)	[36]
OSAVI	OSAVI = 1.16 × (NIR − R)/(NIR + R + 0.16)	[37]
RVI	RVI = NIR/R	[26]
RVI2	RVI2 = NIR/G	[38]

**Table 2:** The R^2^ values predicted by simply using vegetation indices.

	Growth Stage	Three-Leaf Stage	Emergence Stage	Jointing Stage	Large Flare Opening Stage	Tasseling Stage	Flowering Stage
Model							
LTSM(CIS)		0.524	0.516	0.633	0.649	0.546	0.634
RF(CIS)		0.521	0.509	0.471	0.446	0.537	0.489
PLSR(CIS)		0.376	0.369	0.357	0.401	0.323	0.356

**Table 3:** The R^2^ values predicted using index coefficients and plant height.

	Growth Stage	Three-Leaf Stage	Emergence Stage	Jointing Stage	Large Flare Opening Stage	Tasseling Stage	Flowering Stage
Model							
LTSM(CIS)		0.731	0.732	0.734	0.744	0.721	0.723
RF(PH+CIS)		0.614	0.6	0.687	0.629	0.576	0.602
PLSR(PH+CIS)		0.473	0.453	0.479	0.552	0.395	0.473

## Data Availability

Data are contained within the article.
